# Strategies in Gene Therapy for Glioblastoma

**DOI:** 10.3390/cancers5041271

**Published:** 2013-10-22

**Authors:** Aneta Kwiatkowska, Mohan S. Nandhu, Prajna Behera, E. Antonio Chiocca, Mariano S. Viapiano

**Affiliations:** Department of Neurosurgery, Brigham and Women’s Hospital and Harvard Medical School, Boston, MA 02115, USA; E-Mails: akwiatkowska@partners.org (A.K.); nmohansobhana@partners.org (M.S.N.); pbehera@partners.org (P.B.); eachiocca@partners.org (E.A.C.)

**Keywords:** gene therapy, oncolytic virus, stem cells, nanotechnology, gene transfer, glioma invasion, suicide gene, immunomodulation

## Abstract

Glioblastoma (GBM) is the most aggressive form of brain cancer, with a dismal prognosis and extremely low percentage of survivors. Novel therapies are in dire need to improve the clinical management of these tumors and extend patient survival. Genetic therapies for GBM have been postulated and attempted for the past twenty years, with variable degrees of success in pre-clinical models and clinical trials. Here we review the most common approaches to treat GBM by gene therapy, including strategies to deliver tumor-suppressor genes, suicide genes, immunomodulatory cytokines to improve immune response, and conditionally-replicating oncolytic viruses. The review focuses on the strategies used for gene delivery, including the most common and widely used vehicles (*i.e.*, replicating and non-replicating viruses) as well as novel therapeutic approaches such as stem cell-mediated therapy and nanotechnologies used for gene delivery. We present an overview of these strategies, their targets, different advantages, and challenges for success. Finally, we discuss the potential of gene therapy-based strategies to effectively attack such a complex genetic target as GBM, alone or in combination with conventional therapy.

## 1. Introduction

Glioblastoma (GBM) is the most aggressive tumor of the Central Nervous System (CNS) and its prognosis is one of the worst among all cancer types. Although the number of GBM cases is small compared to other solid tumors, population statistics still reveal a dim picture after years of research and improvement in the clinical management of this disease [1]. Of the approximately 13,000 new patients diagnosed with GBM in the US every year, almost 50% of the patients die within one year and 90% within three years following diagnosis [2], causing more years of life lost than most types of cancer [3]. 

GBM is therefore considered a fatal malignancy, incurable by conventional therapeutic strategies [4]. A major factor that contributes to the dismal prognosis of GBM is the physical and physiological isolation of these tumors within the CNS, which makes difficult the delivery of chemotherapeutics. In addition, the CNS is largely regarded as an immune sanctuary protected from systemic immune responses, therefore facilitating immune evasion of tumor cells and limiting the efficacy of systemic immune-boosting approaches. A third and critical factor that makes these tumors extremely difficult to eradicate is the highly invasive nature of GBM cells, which disperse along blood vessels and white matter, resulting in a disseminated disease that is impossible to completely resect [4]. Finally, the presence of a disseminated tumor stem-like cell population that supports tumor self-renewal and is particularly resistant to chemo- and radio-therapy, is another major factor underlying tumor recurrence and poor long-term survival [5]. 

Given the resistance of these tumors to conventional therapeutic approaches there is an urgent need to develop alternative strategies to complement or improve current approaches and improve long-term patient survival. Strategies under development include novel adjuvant chemotherapeutics to be combined with standard care, as well as novel molecularly-targeted approaches against the tumor and its microenvironment. In this review we will focus on a host of molecularly-targeted approaches collectively aggregated under the concept of *gene therapy*.

Gene “therapy” as the possibility of selecting the genetic information of organisms was first mentioned even before the identification of DNA as genetic material [6], but the formal concept of gene therapy as horizontal transfer of genetic material with the potential to treat diseases only solidified in the early 1970s [7], when technological advances allowed researchers to deliver tailored genetic material to mammalian cells. “Strict” gene therapy is based on the ability to replace a defective gene function through delivery and integration of the functional version of the gene. Therefore, gene therapy strategies have largely been developed for genetic diseases with clear dependency on a single gene deficiency [8], such as recessive enzymatic deficiencies and blood disorders. 

Despite being a genetic disease, the possibility of applying strict gene therapy for cancer is less straightforward since tumors develop through multiple known and unknown genetic abnormalities. Moreover, the accumulation of mutations and evolution of the tumor’s genetic makeup during malignant progression make cancers a genetic moving target that would defeat the single gene-replacement approach. Therefore, the concept of gene therapy for cancer has been widened to encompass the general delivery of therapeutic genetic material to the tumor, to kill cancer cells or enhance the immune response against them.

**Figure 1:**
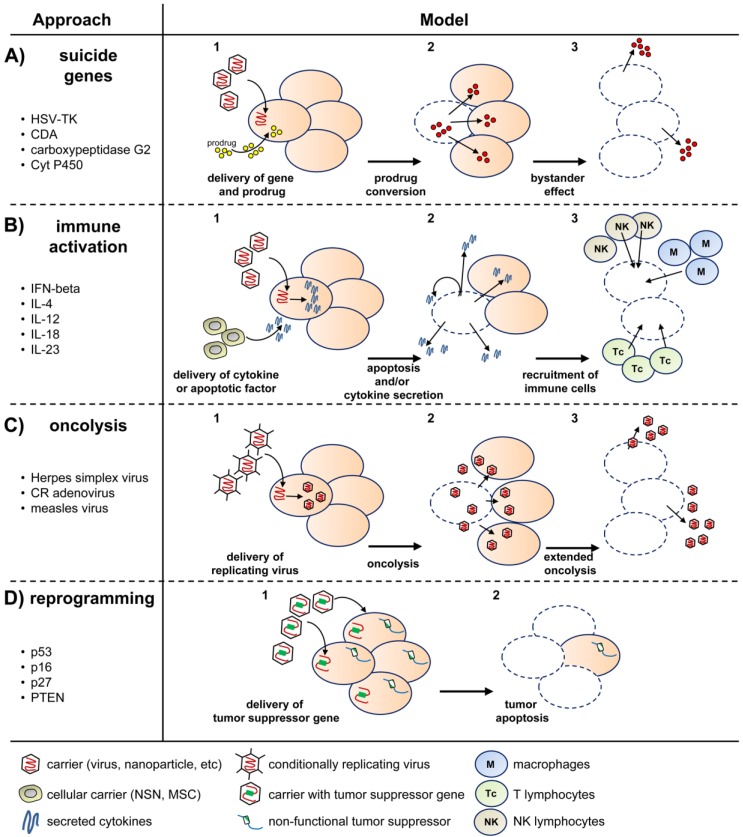
Different strategies for gene therapy of GBM. (**A**) Suicide genes: GBM cells receive the suicide gene by local injection of a carrier, together with systemic delivery of a prodrug (1). The suicide gene converts the prodrug into a cytotoxic product (2) that kills the recipient cell and non-transduced bystander tumor cells (3); (**B**) Immune activation: The gene for an immunomodulatory cytokine is delivered to the tumor cells using several possible vehicles with tumor tropism (1), including viruses or stem cells. Cytokine expression increases tumor cell apoptosis (2) and activates immune cells such as macrophages, natural killer cells or T-cell lymphocytes (3); (**C**) Oncolysis: The tumor is infected with conditionally-replicating oncolytic viruses (1) that lyse the tumor cells (2–3) while sparing normal ones; (**D**) Reprogramming: Tumors receive the functional copy of a tumor suppressor gene (1), which subsequently induces cell cycle arrest or apoptosis (2).

Strategies for gene therapy of cancer in general, and gliomas in particular, have been in development for the past twenty years, with a strong record of success in pre-clinical models and an increasing number of models reaching clinical trials [9] (see Table 1 for a summary of active trials). Major approaches employed for gene therapy of GBM have included: (1) delivery of suicide genes to convert prodrugs in the tumor and achieve tumor cell death; (2) delivery of cytokine genes to activate and attract immune cells against the tumor; (3) delivery of tumor-suppressor genes to reprogram tumor cells into apoptosis; and (4) delivery of conditionally-replicating viruses to specifically lyse tumor cells while sparing normal tissue. Carriers of genetic material have usually been viruses, but alternative vehicles such as stem cells, nanoparticles and liposomes, have also been extensively developed and reached the clinical stage. The following sections will describe these approaches in detail, comparing the advantages and specific challenges faced by each one. A summary of these strategies and examples of representative genes employed are shown in Figure 1. 

**Table 1:** Active clinical trials for gene therapy of GBM. Clinical trials listed in this table are registered with *active* status (open, recruiting or ongoing) as of May 2013. Source: US National Institutes of Health [10] and Journal of Gene Medicine [11].

Country/Identifier	Model	Strategy/goals	Carrier	Phase
US/NCT00589875	AdV-TK	Suicide gene	non-replicating virus	IIa
China/CT00870181	AdV-TK	Suicide gene	non-replicating virus	II
US/NCT00634231	AdV-TK (plus radiotherapy)	Suicide gene	non-replicating virus	I
US/NCT00751270	AdV-TK (plus radiotherapy)	Suicide gene	non-replicating virus	Ib *
US/NCT00589875	AdV-TK (plus radiotherapy)	Suicide gene	non-replicating virus	IIa *
US/NCT01811992	(1) AdV-hCMV-TK and	(1) Suicide gene	non-replicating virus	I
	(2) AdV-hCMV-Flt3L	(2) Immune stimulation		
US/NCT01156584	retroviral vector (Toca-511) carrying CDA	Suicide gene and viral oncolysis	replicating virus	I/II
US/NCT01174537	New Castle Disease Virus	Viral oncolysis	replicating virus	I/II
US/NCT01301430	H-1 parvovirus (ParvOryx-01)	Viral oncolysis	replicating virus	I/II
US/NCT01491893	engineered chimeric poliovirus (PVS-RIPO)	Viral oncolysis and immune stimulation	replicating virus	I
US/NCT00390299	Engineered measles virus (MV-CEA)	Viral oncolysis and immune activation	replicating virus	I
US/NCT01582516	AdV-Delta-24-RGD	Viral oncolysis	replicating virus	I/II
	delivered via CED			
US/NCT00805376	AdV-Delta-24-RGD-4C	Viral oncolysis	replicating virus	I
UK/UK-0050	HSV 1716	Viral oncolysis	replicating virus	II
US/NCT01172964	stem cells carrying CDA	Suicide gene	neural stem cells	Pilot

*Abbreviations*: AdV, adenovirus; CDA, cytosine deaminase; CED, convection-enhanced delivery; CMV, cytomegalovirus promoter; Flt3L, FMS-like tyrosine kinase 3 ligand; TK, thymidine kinase; *: E.A.C. is currently involved in these two active (non-recruiting) clinical trials.

## 2. Virus-Based Gene Therapy of GBM

Viruses targeting mammalian cells have evolved as effective vehicles for horizontal gene transfer and have therefore been the preferred approach for gene therapy since its beginnings [7]. Moreover, the specific neurotropism of certain herpesviruses, adenoviruses and paramyxoviruses [12,13,14,15,16] has made them particularly valuable to target cells of the neural lineage, including malignant brain tumor cells. The first attempt to treat gliomas with a non-engineered virus was an unsuccessful study using attenuated mumps virus, conducted in 1982 [17]. A decade later, two improved strategies using engineered viruses were described almost simultaneously: the use of transduced cells delivering engineered retroviruses into the tumor stroma [18,19], and the first use of an engineered Herpes simplex virus (HSV) for selective replication in glioma cells [20]. This research marked the beginning of two of the major strategies used in glioma virotherapy: (a) targeting the tumor with replication-deficient viruses carrying conditionally-expressing suicide genes; and (b) using tumor-specific, replication-competent oncolytic viruses. The following sections describe the major strategies that have been employed for GBM gene therapy using viruses and their current pre-clinical and clinical status. 

### 2.1. Viral Delivery of Suicide Genes

Systemic chemotherapy of tumor cells is usually limited by toxic side effects caused on dividing normal cells. Suicide gene therapy was envisioned as a way to overcome this limitation, and is based on the systemic delivery of an inactive prodrug together with tumor-specific expression of a drug-activating enzyme (the *suicide gene*) [21,22]. Suicide genes are usually absent or expressed at very low levels in mammalian cells [23] and are therefore delivered using a viral transduction system [9].

The best studied suicide gene is the HSV-derived enzyme Thymidine Kinase (HSV-TK) [9,24,25]. This enzyme catalyzes the phosphorylation of cytotoxic nucleoside analogues that can be incorporated into the DNA of actively proliferating cells, disrupting DNA replication and halting cell division. Since the prodrug nucleosides are poor substrates for mammalian TK, the toxic effect can be restricted to actively dividing cells that have been transduced with HSV-TK using non-replicating herpesvirus or adenovirus [9,23]. This strategy was first used by Ezzedine and colleagues in 1991 to demonstrate the selectivity and efficacy of HSV-TK to kill subcutaneously-implanted glioma cells upon administration of the prodrug ganciclovir [26]. Improvements of this strategy have included the use of different drug formulations to enable sustained intratumoral drug delivery [27] and the use of mutant HSV-TK versions that confer increased sensitivity to the antiviral prodrugs [25,28].

An added advantage of suicide gene therapy is the spread of cytotoxicity from the originally infected cells to neighboring neoplastic, non-infected cells, an effect known as *bystander cytotoxic effect* [22,29,30]. In the case of TK, however, the bystander effect is somewhat limited because phosphorylated nucleoside analogues do not cross the cell membrane. Instead, they must be transferred to the neighboring cells via gap-junctions or by release of apoptotic vesicles from the infected, dying cell [31,32,33]. 

In spite of the initial promise of suicide gene therapy for glioma, further evidence suggested that even HSV-TK expressing cells could become resistant to the prodrugs, therefore requiring combination of this molecularly-directed gene therapy with conventional chemo-radiotherapy [25,28,34,35,36]. There has also been concern about possible toxic effects, poor rate of delivery of HSV-TK to the tumor cells [37], and immune response against the delivery vehicle. For example, non-human primates treated with adenovirus-delivered HSV-TK showed dose-dependent toxicity and developed antibodies against the viral particles [38]. Chronic inflammatory symptoms (including macrophage activation and lymphocyte infiltration) were also observed in the brain of long-term surviving rats that had been implanted with intracranial gliomas and treated with adenovirus-delivered HSV-TK [39]. Despite these caveats, viral-delivered HSV-TK has proven to be a safe strategy in multiple phase I and II clinical trials [40,41,42] and continues to be the most common suicide gene approach in active trials (see Table 1 for active clinical trials). Side effects have been fairly minimal and the major limiting factor has not been toxicity but lack of significant improvement in efficacy against placebo. This was demonstrated in a large, multicenter, phase III clinical trial for HSV-TK (GLI328 International Study Group) that employed retrovirus-producing cells to deliver HSV-TK gene therapy in patients with newly-diagnosed GBM [37]. The trial reported a good safety profile for this adjuvant treatment, although there were no significant improvements in progression-free or overall survival. This lack of effect was largely attributed to poor distribution of the carrier and limited delivery of HSV-TK into the tumor. 

Another widely studied suicide gene is the bacterial enzyme Cytosine Deaminase (CDA), which converts the prodrug 5-fluorocytosine to the toxic compound 5-fluorouracil (5-FU). 5-FU can be further converted to 5-fluorouracil triphosphate, which interferes with RNA processing, or 5-fluorouridine-5'-monophosphate, which irreversibly inhibits DNA synthesis [23]. Importantly, 5-FU can diffuse to neighboring cells and achieves bystander cytotoxic effect that does not require the presence of physical cell-cell contacts [21,43]. Further enhancement of cytotoxicity has been achieved by using an engineered bacterial CDA (bCDA-Asp^314^Ala) with increased affinity for 5-fluorocytosine. Combination of this recombinant CDA with radiotherapy has shown significant tumor cell killing and delayed tumor growth in xenograft models of glioma [23]. Adenovirus-delivered CDA has also been combined with a second enzyme, Uracil Phosphoribosyl Transferase (UPRT), which catalyzes the conversion of 5-FU into 5-fluorourydine-5'-monophosphate. Simultaneous expression of CDA and UPRT genes has shown cooperative antitumor effects [41]. Interestingly, the sensitivity of glioma cells to the combination of CDA and UPRT plus systemic 5-fluorocytosine seemed to be p53-dependent [44], suggesting that p53 status could be used as stratification criteria for this treatment. Viral-delivered, CDA-based therapy has reached the clinical stage and a non-lytic, replicating retroviral vector (Toca-511) [45] is currently being tested in a phase I/II clinical trial to deliver the enzyme in combination with 5-fluorocytosine in patients with recurrent high-grade glioma (NIH trial NCT01156584, Table 1). 

A third example of nucleoside-modifying suicide gene therapy is the use of *E. coli*-derived Purine Nucleoside Phosphorylase (PNP), which can convert non-toxic adenosine ribonucleosides (e.g., fludarabine) into toxic adenine analogs (2-fluoroadenine) that disrupt RNA processing. These metabolites can diffuse to neighboring cells, resulting in robust bystander effect in proliferating and non-proliferating cells [46,47]. Retrovirus-carried PNP has been shown to integrate in the host cell DNA, leading to long-term effect of this treatment *in vivo* [47]. An important development of this approach was the combination of herpesvirus-delivered PNP with antibiotics to remove intestinal flora that could convert the prodrug outside the tumor. This approach allowed the use of lower doses of the cytotoxic agent, enhancing chemoprotection and efficacy in a mouse model of glioma [48]. 

Nucleic acid-targeting gene therapies have also used transgenes coding for enzymes that generate DNA-alkylating compounds. This strategy has a considerable advantage because cytotoxicity does not depend on DNA replication or RNA expression, therefore killing both proliferating as well as quiescent glioma cells. An example of pro-alkylating suicide gene that has been tested in several solid tumor models is the bacterial enzyme carboxypeptidase G2 (CPG2). When combined with nitrogen mustards prodrugs (such as 4-[(2-chloroethyl)(2-mesyloxyethyl)amino]benzoyl-l-glutamic acid (CMDA) or ZD2767P), CPG2 yields mustard-like compounds that crosslink DNA [49]. This conversion is specific to CPG2, which has no mammalian homologues, therefore preventing non-specific generation of highly toxic alkylating compounds in off-target tissues. Engineering of this enzyme for cell-surface expression has been used to enhance the bystander effect of prodrugs that cross the cell membrane poorly and do not reach therapeutic effect with intracellular CPG2 [50]. Although CPG2 has not been tested in gliomas *in vivo*, it has been delivered to cultured glioma cells using replication-deficient adenoviruses and shown cytotoxicity comparable or higher than HSV-TK in the same cells [51].

One extensively studied pro-alkylating suicide gene therapy has used cytochrome P450 (*CYP2B1* gene), the only example of a mammalian-derived suicide gene. This cytochrome can hydroxylate the immunomodulatory prodrug cyclophosphamide (CPA), generating an alkylating phosphoramide mustard. The first demonstration of this strategy for gliomas *in vivo* used fibroblasts transduced with a replicating retrovirus carrying CYP2B1, which were injected intratumorally in mice carrying intracranial GBM xenografts [52]. Following intrathecal administration of CPA, this study demonstrated partial regression of the intracranial tumor mass and limited or absent tumor dispersion to the meningeal space. Further development of this strategy placed the CYP2B1 gene in a replicating HSV (see description of oncolytic HSV in Section 2.5), showing strong antitumor effect when CPA chemotherapy was combined with HSV-mediated tumor oncolysis [53]. A significant advantage of CPA is the inhibitory effect of this drug on the innate immune activity against HSV, therefore reducing viral clearance and enhancing both gene delivery and viral oncolysis [54]. 

Finally, it should be noted that the concept of suicide genes can be expanded to include additional examples of cytotoxic genes as long as they are specifically delivered to and active in the target tumor cells. One example is the potent *Pseudomonas* exotoxin A, a cytotoxin produced by *Pseudomonas aeruginosa* that disrupts protein synthesis. A truncated form of this toxin conjugated to mutated interleukin IL-13 (mhIL-13-PE, marketed under the name Cintredekin Besudotox) specifically binds and kills glioma cells while sparing normal neural cells that lack the glioma-specific receptor for the interleukin (IL13Rα2) [55]. In an elegant development of this strategy, an adenoviral vector was developed for conditional expression of mhIL-13-PE in transduced glioma cells [56]. The vector also expressed IL-4 to saturate the normal receptor of IL-13 (IL4R/IL13R), therefore preventing binding of the chimeric toxin to normal neural cells and achieving specific cytotoxicity. Inoculation of this virus in multiple models of intracranial glioma using athymic and immunocompetent mice resulted in significant tumor toxicity and increased animal survival with high proportion of long-term survivors [56]. 

### 2.2. Viral Delivery of Tumor-Suppressor Genes

Tumor suppressor genes are major regulators of DNA repair, cell proliferation, and apoptosis. Deletions and inactivating mutations in those genes are common in all cancers, including gliomas. In particular, mutations in three pathways containing tumor suppressors are commonly associated with high-grade human gliomas: p53/MDM2, p16/Rb, and PTEN [57]. Based on this feature of brain tumor biology, viral strategies have been designed in an attempt to reprogram tumor cells by restoring tumor suppressor activity in cells carrying inactivating mutations in those genes. 

p53 is considered one of the most critical mediators of growth arrest and apoptosis in response to DNA damage, hypoxia, and growth factor withdrawal. This is one of the most frequently mutated genes in gliomas, being inactivated in about 30% of primary and 65% of secondary GBMs [58]. Tumor suppressor therapy using p53 in glioma cells was first attempted by delivering this gene, under control of the potent CMV promoter, using non-replicating adenovirus serotype 5 (Ad5CMV-p53). Restoration of the functional gene induced robust apoptosis of the infected cells *in vitro* and reduced tumorigenesis *in vivo* [59]. However, inoculation of the same vector in GBM-bearing mice was insufficient to reduce intracranial tumor growth [60], possibly due to poor gene transfer in established tumor tissue. Interestingly, adenoviral transduction of p53 into wild type p53-bearing glioma cells has also shown marginal [59,60,61] to robust [62] inhibition of cell proliferation and induction of apoptosis, indicating that these effects are not solely dependent on the restoration of a functional copy of p53. 

Limitations of tumor suppressor-based gene therapy have included poor gene transfer as mentioned, lack of bystander effect, and potential resistance arising from the inherent genetic heterogeneity within GBM. However, transduction of tumor suppressor genes such as p53 may present an excellent opportunity for combinatorial therapy since they could re-sensitize the cells to radiation and chemotherapy [59,61,63,64] or reduce immune evasion when combined with immune-boosting strategies [65]. Successful pre-clinical results with p53-restoration led to a phase I clinical trial of Ad5CMV-p53 (INGN 201) for recurrent malignant glioma, involving injection of the virus pre- and post-resection. Exogenous p53 protein was found in the nuclei of tumor cells in all patients treated with this strategy, although transduced cells were found only within a short distance from the injection site. Adverse events were minimal and the trial tested doses up to 3 × 10^12^ plaque-forming units (p.f.u.) without reaching maximum tolerable dose [66].

p16^INK4A^ is another major tumor suppressor that causes cell cycle arrest at the G1-S transition point by maintaining hypo-phosphorylated status in the Retinoblastoma protein (Rb) [59,60]. Adenoviral-mediated restoration of p16 in GBM cells induced, as expected, tumor cell cycle arrest in G1-S phase [67,68]. Surprisingly, this overexpression of p16 also caused an unexpected reduction in GBM cell invasion, resulting from decreased activity of matrix metalloprotease 2. This important anti-invasive effect is remarkable since it was not observed after restoration of the tumor suppressor p21/WAF, which prevents G1-S transition by a molecular mechanism similar to p16 [67]. 

The third major tumor suppressor regulating glioma growth and invasion is the Phosphatase and Tensin Homologue (PTEN), which is lost, mutated or inactivated in 40%–50% of all gliomas (~25% of primary GBMs) [58], resulting in high levels of dys-regulated PI3K activity and downstream signaling [69]. Adenoviral re-expression of PTEN in GBM cells inhibited Akt kinase activity, leading to tumor cell apoptosis [70]. Infection with this virus was also shown to decrease metalloprotease expression and glioma cell invasion *in vitro* [71]. When tested in GBM-bearing mice, adenoviral restoration of PTEN has shown important effects on the tumor microenvironment, inducing an anti-angiogenic response even in presence of pro-angiogenic stimuli such as loss of p53 or presence of constitutive EGFR activity [72]. 

Another important example of viral-delivered tumor suppressor strategy has been demonstrated with p27, an inhibitor of Rb phosphorylation that arrests the cell cycle in G1. p27 levels are regulated by complex feedback loops involving phosphorylation of this protein in Thr^187^ and further proteasomal degradation [73]. p27 activity was restored in GBM cell lines and GBM-derived primary cells using adenovirus to carry either wild type (Ad-p27wt) or a degradation-resistant Thr^187^-mutant (Ad-p27mt, Thr^187^Ala). In all cases, recovery of functional p27 promoted Rb dephosphorylation, apoptosis, and suppression of tumor growth [68]. Interestingly, while p27wt arrested the cell cycle in G1-S transition as expected, p27mt did so at the G2-M checkpoint by undefined mechanisms that were not observed in other cell types. Additional studies using adenoviral-restored p27 have demonstrated that this tumor suppressor reduces GBM growth *in vivo* as well as local invasion and tumor-induced neo-angiogenesis, with these effects being caused by a cytoskeletal anti-migratory effect of p27 both in GBM cells and tumor-associated endothelial cells [74]. 

### 2.3. Viral Delivery of Immunomodulatory Genes

The CNS is relatively isolated from systemic immune responses and is therefore difficult to induce the immune system to mount an effective local anti-tumor response against gliomas [42]. This difficulty is increased by the ability of glioma cells to suppress and effectively evade cellular immune responses [9]. In order to promote effective immunotherapy against glioma, viruses have been engineered for targeted delivery and expression of cytokines that activate and recruit immune effectors to the tumor. 

An excellent example of this strategy was the early use of a replication-deficient adenovirus carrying the gene for the potent immune-boosting cytokine interferon beta (IFN-beta) under control of the CMV promoter [75,76]. Using this vector, IFN-beta was expressed in pre-established, subcutaneous gliomas in nude mice, resulting in enhanced immune cellular response against the tumor (lymphocyte infiltration), tumor regression, and significantly prolonged animal survival [76]. This strategy reached a phase I clinical trial for recurrent malignant glioma, where an IFN-beta-expressing, non-replicating adenoviral vector was stereotactically injected in the tumor before surgical resection [77]. The trial demonstrated that the virus inoculation was safe and well tolerated, while analysis of the resected tumors demonstrated dose-dependent induction of local inflammation and tumor necrosis.

Using recombinant parvoviruses, another immunomodulatory strategy was attempted by simultaneous delivery of IFN-gamma-inducible protein 10 (CXCL10) and TNF-alpha in a syngeneic mouse model of GBM [78]. Results showed synergistic activity of both vectors and complete regression of tumors generated from cells that had been transduced with both cytokines before implantation. Multiple mechanisms were proposed to contribute to this synergy, including CXCL10-mediated recruitment of activated T and NK lymphocytes to the tumor, inhibition of tumor angiogenesis by CXCL10, and TNF-alpha-mediated tumor necrosis and maturation of dendritic cells. Despite these exciting results, the effect of the viruses in naïve pre-established tumors was marginal, resulting in delayed tumor growth but no regression [78].

Viral-mediated delivery of other interleukins has not been as extensively exploited in GBM as in other cancers, but work in this direction has definitely shown therapeutically relevant results. Non-replicating adenoviral-associated virus (AAV) and replicating HSV have been employed to deliver IL-12 in experimental models of GBM [79,80,81], resulting in local immune mechanisms such as increased IFN-gamma expression, microglial activation, and recruitment of T and NK lymphocytes, with a significant antitumor effect. 

### 2.4. Viral Delivery of Genes That Modify the Tumor Stroma

**Figure 2:**
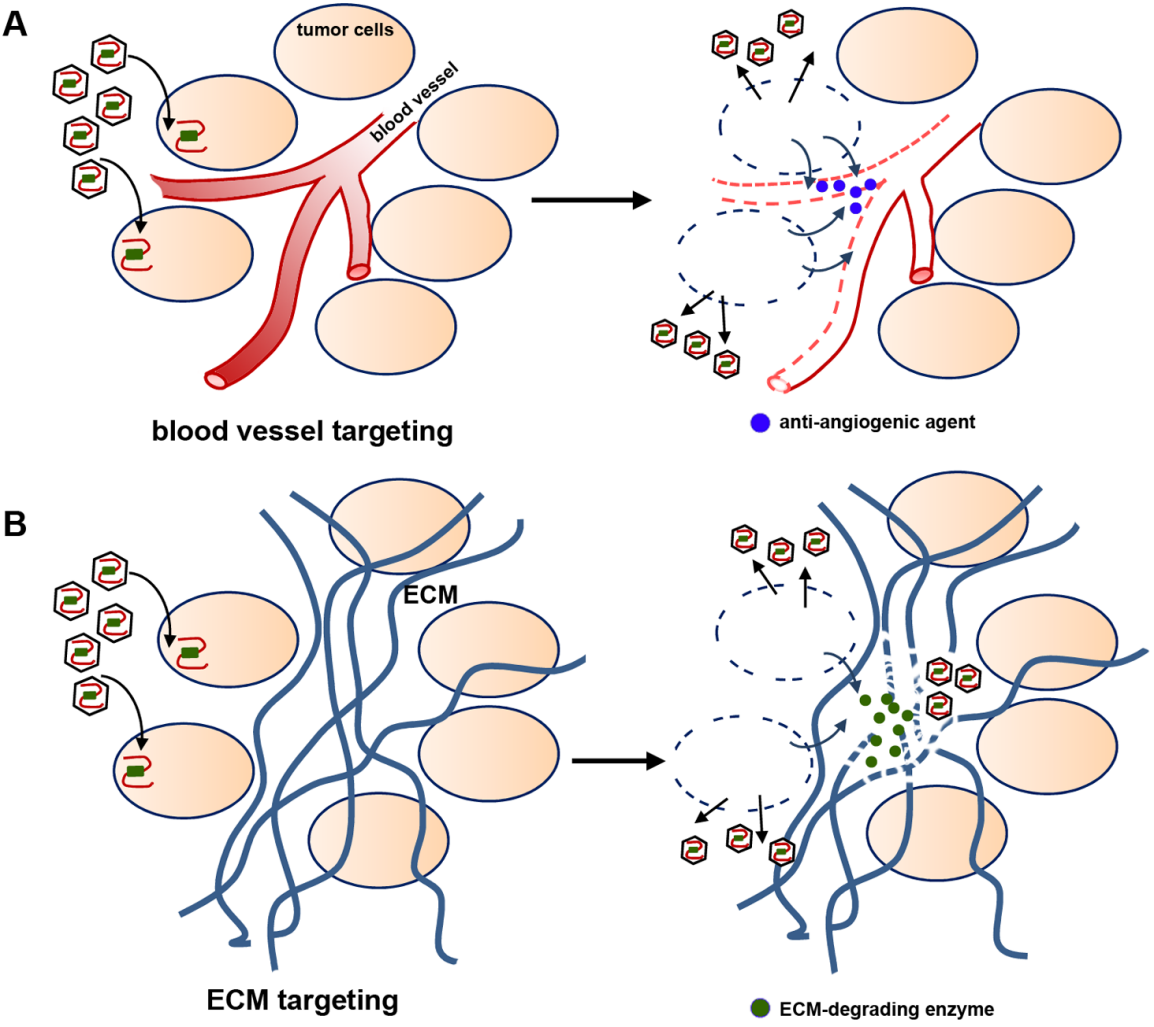
Strategies targeting the GBM microenvironment. To enhance viral oncolysis conditionally-replicating oncolytic viruses may also carry genes that modify the tumor microenvironment. (**A**) Anti-angiogenic strategies: viruses carry anti-angiogenic factors that reduce vascular support of the spared tumor not reached by oncolysis; (**B**) Anti-ECM strategies: viruses carry enzymes that degrade ECM components, increasing dispersion of viral particles and oncolytic efficacy.

The gene-delivery strategies described in the previous sections (as well as viral-mediated oncolysis, in the following section) target specifically the tumor cells for immediate cell death. Additional effects such as reduced tumor vascularization and invasion may be observed (and welcomed) but are not usually part of the design rationale. However, viruses can also be engineered to deliver genes that specifically affect the tumor microenvironment. Two clear examples of this strategy are viruses carrying anti-angiogenic genes or genes that remodel the tumor extracellular matrix (as illustrated in Figure 2). 

Initial attempts to specifically inhibit glioma angiogenesis with gene therapy involved the intratumoral injection of retrovirus and AAV carrying the antiangiogenic factor angiostatin [82,83]. A subsequent study, using systemic instead of local delivery, followed a similar approach with adenoviral-delivered endostatin [84]. In all cases tumor vascularization was significantly inhibited and tumor growth was reduced more effectively than with the parental viruses. A more recent approach has combined anti-angiogenesis with viral oncolysis using conditionally-replicating oncolytic HSV (see details of this virus in the following section). Two oncolytic HSVs were engineered to express the anti-angiogenic protein vasculostatin under control of the promoter for the early viral gene IE4/5 [85,86]. Secretion of vasculostatin was detected a few hours after infection of glioma cells and results *in vivo* with both viruses showed remarkable reduction in microvessel density, tumor perfusion, and overall tumor progression. Coupled with their oncolytic ability, these antiangiogenic viruses offered significantly better antitumor efficacy when compared with their parental HSV strain. 

Delivery of genes that can remodel the tumor extracellular matrix (ECM) has been rarely attempted, despite the fact that this matrix is the most immediate physical barrier to viral dispersion and a significant factor that limits viral effects to short distances from the inoculation site [87]. Indeed, pre-injection of proteases that can degrade tumor ECM proteins enhances subsequent viral spread and infection [88]. Following this rationale, a conditionally-replicating oncolytic HSV was engineered to express the bacterial enzyme chondroitinase ABC-I, which degrades major components of the glioma ECM such as hyaluronan and chondroitin sulfate proteoglycans [89]. The resulting virus demonstrated increased efficacy against the tumor compared to parental HSV and, as expected, dispersed farther away from the sites of inoculation. Local degradation of ECM was also demonstrated, which, importantly, did not enhance the invasive ability of the remaining tumor cells. 

### 2.5. Replication-Competent Oncolytic Viruses

Oncolytic virotherapy (OV) can be considered a gene therapy approach where the whole virus becomes the genetic payload. OV utilizes replication-competent viruses that can infect and lyse tumor cells, with or without concomitant gene transfer [9,90,91]. OV strategies exploit two major properties of the viruses used as vehicles/lytic agents: Tropism towards cells of neural lineage/brain tumor cells, and the ability to specifically replicate in tumor cells with altered signaling pathways while sparing normal cells [90,92,93,94]. Oncolytic HSV, conditionally-replicating adenovirus (CRAd), reovirus, poliovirus, engineered retroviruses, Newcastle Disease virus and measles virus have been evaluated for OV therapy of GBM [95].

HSV is an enveloped DNA virus with wide tropism that replicates in dividing and non-dividing cells [9,91]. This virus establishes latent infection in post-mitotic neurons and is especially suitable for therapeutic strategies in the CNS. To reduce neurovirulence, engineered versions have been attenuated through deletions in genes necessary for viral replication in normal cells, such as the ribonucleotide reductase ICP6 (*UL39*) and the protein synthesis promoting factor ICP34.5 (*γ34.5*) [96]. Two recombinant oncolytic HSV have reached clinical trials for recurrent high-grade glioma: The virus HSV G207 (with an inactivating insertion in ICP6 and deletions in both copies of γ34.5) was tested in a phase I clinical trial where no dose-limiting toxicity was observed up to the maximum tested dose of 3 × 10^9^ p.f.u. The study reported eight patients (out of 21) with radiographic/histologic response to the treatment and two long-term survivors [97]. A further phase Ib trial demonstrated the safety of multiple dose delivery of the same virus, including inoculation both in the pre-resected tumor and the post-resection cavity [98]. A similar virus, HSV1716 (deleted in both copies of γ34.5) reached phase II trial for intratumoral delivery. HSV1716 treatment showed positive response in three out of twelve patients and did not cause toxicity even when patients developed antibodies against the virus [99,100]. A disadvantage of these models is the required deletion of viral genes (γ34.5), which attenuates viral replication and limits the virus efficacy [101]. A new generation of oncolytic HSVs is therefore being studied in pre-clinical models and prepared for clinical trials. A major modification that enhances the efficacy of these viruses is the conditional re-expression of viral genes under promoters that are overexpressed in brain tumor cells compared to normal cells, such as Nestin, GFAP, or survivin [102,103,104].

CRAds with deleted viral genes have been employed as alternative to oncolytic HSV. Adenoviruses are non-enveloped DNA viruses that infect proliferating and quiescent cells and can integrate, with low frequency, into a defined region of the host genome [9,91]. Two extensively studied CRAds in glioma are ONYX-015 and Ad5Delta24, both of which target cells with dys-regulated signaling pathways. ONYX-015 has a deletion in the gene coding for the viral protein E1B that binds and inactivates p53. Due to this deficiency, the virus was originally expected to replicate selectively in p53-deficient cells (*i.e.*, tumor cells) [105], although it was later shown that its oncolytic activity in gliomas was independent of p53 status and even increased in wild-type p53 glioma xenografts [106]. ONYX-015 was tested in a phase I trial for recurrent malignant gliomas, being injected only in the post-resection cavity [107]. Although the study could not demonstrate a significant antitumor efficacy, it showed absence of serious adverse effects and good tolerance to the virus, without reaching maximum tolerable dose even at 10^10^ p.f.u. 

Similarly to ONYX-015, Ad5Delta24 has a genetic deletion for the viral protein E1A, which inactivates the Rb tumor suppressor. Ad5Delta24 therefore replicates in glioma cells with a deficient Rb pathway, causing significant growth inhibition of xenografted tumors in mice [108]. This virus was further engineered by introducing the integrin-binding RGD motif in the knob domain of the viral fiber protein (Ad5Delta24-RGD) [109], which enhanced the specific targeting of tumor cells and increased oncolytic efficacy against gliomas [110]. Ad5Delta24-RGD is currently being tested in a phase I clinical trial for recurrent malignant glioma (NIH trial NCT00805376, Table 1) [111]. 

A novel CRAd that has been recently developed replaces the strategy of viral gene deletion by using instead the promoter of a gene highly expressed in glioma cells (survivin) to drive the expression of E1A [112]. This virus (CRAd-survivin-pK7) has in addition a poly-lysine sequence added to the fiber knob, which enhances the binding to cell surface proteoglycans and increases viral entry and anti-tumoral efficacy against differentiated as well as stem-like glioma cells [112,113]. 

In addition to the extensively used HSV and CRAds, other viruses have been engineered for OV of glioma, including measles, vaccinia, rhabdoviruses and polioviruses [114,115]. Of these, the measles paramyxovirus (MV, attenuated Edmonston strain) was the earliest to be considered as a potential oncolytic agent since initial reports in the 1970s suggested that measles infection was associated with regression of lymphoma and leukemia [116,117]. MV targets cells that express the membrane receptors CD46 and Signaling Lymphocyte Activating Molecule (SLAM), inducing the formation of multinucleated syncytia followed by apoptosis. MV variants have been engineered to express IL-13 [118] or a single-chain antibody against the vIII deletion variant of EGFR [119], therefore re-targeting the viruses against proteins highly expressed on the surface of glioma cells and increasing their oncolytic efficacy. Additional engineering of MV to express the circulating carcinogenic embryonic antigen (MV-CEA) has been used to monitor the course and maintenance of MV infection [120]. Toxicological data in macaques has shown absence of neurotoxicity of MV-CEA [121] and supported an ongoing phase I clinical trial (NIH trial NCT00390299, Table 1). Recent data has also shown that MV is highly effective against glioma-derived stem-like cells [122], which makes it an attractive approach against this highly resistant population of tumor cells.

### 2.6. Advantages and Challenges of Viral-Based Gene Therapy

Having evolved for horizontal gene transfer, viruses are the most efficient carrier system to deliver genes to tumor cells. Additional modifications described in this review, such as re-targeting and conditional replication have considerably improved the specificity and efficacy of viral vectors, many of which have reached clinical trials for GBM. Moreover, compared to other particle-like carriers (see Section 4.1, Section 4.2, and Section 4.3 on nanotechnology), viruses induce robust bystander cytotoxic effect, attract cellular immune response towards the infected cells, and can directly kill infected glioma cells by cell lysis. These features absent in other vehicles for gene delivery have made viruses one of the most valuable tools for gene therapy of GBM. 

On the other hand, viral carriers and oncolytic viruses still face considerable challenges for successful long-term therapeutic effects. A major difficulty is the limited spread and persistence of the virus in the tumor tissue, caused by factors such as low efficiency of initial infection, rapid clearance of the viral particles by innate immune cells, and physical barriers that limit particle dispersion [123]. Some of these challenges are being actively addressed through strategies involving viral engineering and combination with other antitumor agents. Major developments in the field include improved re-targeting towards GBM-specific receptors [124], combination with drugs that reduce the immune response to the virus [125], “cloaking” of the virus inside carrier cells (described in the following section), enhanced infusion of viral particles via convection-enhanced delivery, and engineering of viruses to express genes that facilitate their physical dispersion [89], among others. As current limitations are overcome, viral-based approaches (alone or combined with conventional therapy) will remain a major choice for gene therapy of GBM. 

## 3. Stem Cell-Based Gene Therapy of GBM

Together with viruses, cells have been used to deliver genetic material to brain tumors for the past twenty years. Indeed, retroviruses themselves were the first genetic payload delivered by cells injected into the tumor stroma [18,19]. Examples of successful carriers have included fibroblasts and HEK293 cells, both used to deliver replicating viruses, suicide genes (HSV-TK), and anti-angiogenic factors in gliomas [126,127]. A major limitation of these cell types has been their lack of migratory ability inside the tumor, a deficiency that was considered a major cause of therapeutic failure in clinical trials of viruses and suicide genes delivered by cells [37]. Currently, the most widely employed cellular carriers are stem cells (SCs) of neural, mesenchymal or embryonic origin. While SCs have been studied for only half as long as viral carriers and only recently reached the clinical stage, they have proved one of the most attractive vehicles to combine gene therapy with virotherapy and conventional therapies. The importance of SCs is underscored by a fundamental property absent in other delivery vehicles: their ability to migrate towards the tumor cells even when injected peripherally [128,129]. This critical feature allows them, in principle, to reach the disseminated tumor cells that are characteristic of GBM [128]. As expected, SCs have already been extensively tested as vehicles for most of the approaches described in the previous sections: delivery of suicide genes, oncolytic viral particles, anti-angiogenic factors, and immune-boosting cytokines, among others.

### 3.1. Neural Stem Cells

Neural Stem Cells (NSCs) are multipotent progenitors of the neural lineage with indefinite self-renewal and ability to differentiate into neurons or glial cells [130]. They are not only highly adapted to the neural environment and architecture [131] but also share many properties (such as cell motility mechanisms [119]) with the elusive glioma stem-like cells. Engineered NSCs were first used against gliomas in 2000 to deliver the cytokine IL-4, therefore improving the immune response against the tumor [132]. Subsequently, they have been largely employed in two major approaches for antitumor gene therapy: as infected carriers of oncolytic viruses or as engineered cells expressing therapeutic genes.

Using NSCs as carriers of oncolytic viruses -which lyse the carrier cell and infect glioma cells- has become an interesting approach with multiple possible advantages over the inoculation of viral particles: migratory NSCs may deliver the viruses at further distances within the tumor compared to virus alone; they can protect the viruses from the host immunosurveillance; and their own lysis removes them from the host after therapy [133]. Following this rationale, Herrlinger and colleagues were the first to show the feasibility of using NSCs to carry conditionally-replicating HSV into pre-implanted cerebral gliomas [134]. Similar studies were further pursued with CRAds [135,136], demonstrating that pre-loading the virus inside NSCs highly enhanced the reach of the virus within the tumor as well as its oncolytic efficacy.

Engineering NSCs to deliver transgenes rather than viruses into the tumor mass has been a more common approach, employed with suicide genes and cytokines. Both CDA and HSV-TK (described in Section 2.1) have been tested in NSC therapy of GBM (e.g., [128,137,138]) and in all cases the use of NSCs has compared favorably against non-migratory cell carriers. NSCs have also been used to carry CDA and IFN-beta cDNAs together, boosting the bystander cytotoxicity with immune response against the tumor and resulting in better antitumor response compared to CDA alone [139]. CDA-carrying NSCs are currently being tested in the first clinical study of feasibility against recurrent high-grade gliomas (NIH trial NCT01172964, Table 1). 

A second group of widely tested transgenes have been immune-boosting interleukins such as IL-4, IL-12 and IL-23 [132,140,141]. These studies led to two important conclusions: First, NSC-mediated sustained delivery of interleukins was found more efficient than viral-based delivery *in vivo* [132] and resulted in improved animal survival. Second, the strong cellular immune response against the tumor (at least in the case of IL-23) resulted in long-term surviving animals resistant to tumor re-challenge, suggesting the possibility of using SC approaches to trigger long-standing antitumor immunity. 

One important cytokine that has been delivered by NSCs in gliomas is the Tumor Necrosis Factor-Related Apoptosis-Inducing Ligand (TRAIL), which is capable of inducing apoptosis in tumor cells with little effect on normal cells. This effect is potentiated in S-TRAIL, a secreted chimeric protein combining the extracellular domains of TRAIL and Flt3L (a ligand for Flt3 tyrosine kinase receptor) [142]. NSCs carrying S-TRAIL were shown to migrate actively towards glioma cells, causing bystander cytotoxicity in the tumor and significantly reducing the burden of pre-implanted gliomas [143]. Moreover, combination of NSCs-S-TRAIL with adjuvant chemotherapy showed strong potentiation of the antitumor effects of temozolomide, PI-103 (a PI3K/mTOR inhibitor), and bortezomib (a proteasome inhibitor) [144,145,146]. 

A third major group of genes delivered by NSCs in glioma models includes anti-angiogenic factors such as endostatin [147], thrombospondin-1 [148], and the angiostatic factor PEX [149]. Although these factors can impair the migration of normal and tumor cells, all the studies reported that active migration of the engineered NSCs towards tumor cells was unaffected. NSCs distributed extensively in the tumor stroma, without concomitant proliferation or differentiation, and caused significant reduction of tumor growth and microvascular density. 

### 3.2. Mesenchymal Stem Cells

Mesenchymal stem cells (MSCs) are non-hematopoietic, adult multipotent stem cells that can be isolated from multiple sources (such as bone marrow, adipose and muscle tissue, or peripheral blood), and expanded with relative ease *in vitro* [150]. These cells migrate to sites of injury and inflammation and are involved in tissue repair. Since tumors behave as non-healing, expanding wounds, they strongly attract MSCs [151]. Based on this strong homing ability, MSCs have been employed in multiple strategies against tumors, including gliomas [152].

As with NSCs, initial studies of MSCs against experimental gliomas involved the overexpression of a therapeutic cytokine (IL-2), resulting in augmented antitumor effect (compared to MSCs alone) and prolongation of animal survival [153]. Due to their easy availability for autologous transplantation, MSCs have become a very attractive alternative to NSCs towards clinical studies, and have already been tested to deliver cytokines, suicide genes, antibody chains and viral particles to gliomas.

Suicide genes originally tested in NSCs have been evaluated in MSCs as well, including HSV-TK [144], CDA [154], and HSV-TK together with connexin-43 to enhance bystander cytotoxicity [145]. MSCs have also been used as vehicle for the suicide gene carboxylesterase [155]. A combination of MSC-delivered carboxylesterase with the prodrug CPT-11 (Irinotecan) was tested in experimental brainstem gliomas and showed effective conversion of the prodrug but only modest improvement in animal survival.

In addition to MSC-delivered IL-2, more recent animal studies have used transduced MSCs to deliver IL-7 [156], IL-12 [157], or IL-18 [158] in glioma xenografts. Strong cellular immune response was observed following infiltration of the MSCs in the tumor stroma, and resistance of the surviving animals to tumor re-challenge was observed in the studies using IL-12 and IL-18. MSCs have also been used to deliver TRAIL constructs, including a shortened form of secretable TRAIL [159] as well as the recombinant TRAIL-Flt3L fusion (S-TRAIL) [160]. Newer studies have combined MSC-delivered TRAIL with radiotherapy [161] and with the anti-inflammatory compound MK886 [162], demonstrating in both cases synergism compared to MSC-TRAIL alone. 

Finally, the use of MSCs as carriers of conditionally-replicating oncolytic viruses such as CRAds has shown that MSCs are capable of suppressing the humoral immune response against the virus, resulting in increased persistence of the viral particles [163]. However, MSCs were found less efficient than NSCs to deliver the same CRAd in orthotopic xenografts and had lesser impact on animal survival [164]. Nevertheless, considerable interest remains in the use of MSCs to deliver those oncolytic viruses that have reached clinical trials, such as Ad5Delta24-RGD described in Section 2.5. MSCs carrying Ad5Delta24-RGD and injected in the peripheral circulation have been shown to reach intracranial gliomas and inhibit tumor growth [165]. Current trends suggest that, if clinical trials with the viruses are promising, the use of SCs to carry them into the tumor could be the next step to improve their therapeutic efficacy. 

### 3.3. Embryonic Stem Cells

Embryonic stem cells (ESCs) are pluripotent cells that form the inner cell mass of the blastocyst during gestation and have unlimited proliferative capacity [166]. Difficulties in obtaining and culturing these cells—both technical and derived from current legislation on SCs—have made their use much more limited than NSC or MSC counterparts, with experiments largely focused on cytokine delivery to gliomas.

The first experiments with mouse ESCs involved engineering to express TRAIL, followed by differentiation into astrocytes using defined culture conditions. ESC-derived astrocytes enhanced apoptosis of co-cultured glioma cells compared to TRAIL alone [167]. Further experiments demonstrated that injection of ESC-derived astrocytes carrying TRAIL could induce severe necrosis in xenografted tumors [168], but no survival studies were pursued. Additional *in vitro* studies using ESC-derived astrocytes demonstrated that delivery of a different cytokine (IL-24) also increased apoptosis of co-cultured glioma cells and potentiated the effects of radiation and temozolomide [169]. 

Human ESC lines have more recently been used to derive NSCs and MSCs, which have been subsequently transduced to deliver the suicide gene HSV-TK [170,171]. The goal of these experiments was to show the successful conversion of ESCs into cells known to have strong tropism for gliomas, and in addition these studies demonstrated successful targeting of intracranial tumor burden and extension of animal survival. 

### 3.4. Advantages and Challenges of Cell-Based Gene Therapy

The use of SCs as gene-delivery vehicles is supported by two unmatched advantages when compared to passive methods of gene delivery: (a) migratory ability that allows them to infiltrate the tumor mass, reaching poorly vascularized areas and the remote borders of the tumor; and (b) strong tropism that attracts them towards glioma cells even when injected peripherally, coupled with ability to cross the blood brain barrier. These two features of SCs, added to the possibility of performing extensive genetic engineering to convert them in carriers of multiple transgenes or whole viral vectors, make them a versatile tool that can be combined with conventional therapy and additional molecular therapy to deliver a large, complex payload inside the tumor.

However, despite their ability to infiltrate gliomas, SCs are essentially neutral and do not have an effect on the tumor unless engineered as gene-delivery vehicles. Since the transgenes are expressed in SCs immediately after transduction (in contrast to viral-carried genes, which are expressed only after infection of the target cells), a first and considerable technical challenge is to ensure that the SCs will survive for as long as it takes to impact the tumor cells, without dying first due to effects of suicide genes or oncolytic viruses [172]. Rapid and efficient delivery to the tumor is therefore a critical factor when SCs are introduced peripherally. Intravenous injection has been the most common route for peripheral introduction of SCs but its efficiency is limited, with less than 2% of the inoculated cells colonizing the tumor [173]. A recent alternative has used intranasal inoculation of NSCs, with a delivery efficiency estimated to be as high as 24% [174]. Additional challenges stem from the choice of SCs in terms of convenience, permanence in the tumor, and therapeutic efficacy. For example, while MSCs are easiest to obtain for autologous therapy, there is active discussion about their relative efficacy compared to NSCs for different gene-therapy strategies [164]. ESCs present, in addition, ethical and regulatory issues for collection and will likely be replaced by induced pluripotent SCs in the future. 

A final and considerable factor that must be addressed with SCs is their safety when introduced in the highly aggressive, cytokine- and growth factor-rich environment of the tumor. To this day studies have shown that none of the different types of SCs employed in animal models suffered neoplastic transformation. However, previous studies have demonstrated that normal neural progenitor cells can contribute significantly to the heterogeneous total mass of PDGF-induced malignant gliomas [175]. Therefore, a desirable feature in future SC-based approaches would be the possibility of selectively eliminating the SCs (e.g., using an inducible suicide gene) after they have reached their therapeutic endpoint. 

Overall, SC-based gene therapy of GBM offers enormous promise and, considering that SCs have become the choice carrier in other neuropathologies, is likely to become the fundamental component of future combinatorial strategies using gene delivery, molecular-targeting therapy and conventional chemoradiotherapy. 

## 4. Nanotechnology-Based Gene Therapy of GBM

The use of nanotechnology, *i.e.*, manipulation of sub-micron-sized materials, to target genetic material into tumor cells is a relatively novel strategy that remains largely experimental. The only nanocarriers that have reached the clinical stage in glioma have been liposomes [9,176], which have long been used as carriers for small molecules in glioma and other cancers. Most nanotechnological approaches for gene therapy have focused in optimizing the DNA-carrying vehicles for effective targeting of tumor cells [177,178], testing many of the same candidate genes used with viruses and SCs. Vehicles that have been recently tested in pre-clinical models include novel formulations of cationic liposomes, nanoparticles, and dendrimers, among others. 

### 4.1. Liposomes

Liposomes are artificial, lipid-based microvesicles usually employed to deliver drugs, peptides and proteins into cells. However, chemical engineering of the lipids also permits the formation of stable DNA-lipid associations that can be exploited to use liposomes as a gene-delivery vehicle. Following this concept, a liposomal vector was devised in the early 2000s to carry a plasmid coding for HSV-TK, which was given to patients with recurrent GBM in a phase I/II trial via intratumoral infusion [179], followed by administration of the prodrug ganciclovir during 14 days. Radiographic response was observed in most patients in this clinical trial, ranging from focal effects to 50% reduction in tumor volume, without major adverse events.

Cationic liposomes have also been used to transfer cytokine genes into glioma cells. A phase I/early phase II clinical trial demonstrated the safety and efficacy of this approach to deliver a plasmid coding for IFN-beta [180] in patients with recurrent malignant gliomas, following resection of the tumor. IFN-beta protein was found in the accumulated fluid in the post-surgical cavity in three out of five patients, for periods of up to ten days after injection. All patients showed positive radiographic response immediately after treatment, although the tumors eventually progressed. A more complex liposome was recently devised to carry both a therapeutic gene (TRAIL) and a cytotoxic drug (paclitaxel), combined with re-targeting via addition of a peptide (angiopep2) that facilitates blood brain barrier crossing. This liposome preparation effectively delivered TRAIL to glioma cells *in vitro* even when protected behind a barrier of normal epithelial cells [181]. More importantly, peripherally delivered angiopep2-liposomes reached intracranially xenografted gliomas in mice, causing local apoptosis in the tumor and extending animal survival. 

Other liposomal preparations have modified not only the surface of the vesicle but also its carrier core. For example, magnetite-core cationic liposomes respond to alternating magnetic fields generating heat. This effect can be used to activate a heat-shock sensitive promoter in the DNA carried by the liposome, thus regulating expression of the therapeutic gene [182]. Using this strategy, Ito and colleagues injected paramagnetic liposomes carrying TNF-alpha under control of the heat-inducible promoter *gadd153* into subcutaneous gliomas implanted in nude mice [183]. Their results showed heat-regulated expression of TNF-alpha protein in the tumor and subsequent retardation of glioma growth. 

A recent and ingenious liposomal design used air-cored liposomes, subsequently loaded with siRNA against the anti-apoptotic protein sirtuin 2 [184]. The liposomes were injected in subcutaneously-implanted gliomas in nude mice, followed by exposure to brief low-frequency ultrasound. This induced cavitation (bursting) of air bubbles in the liposomal core, damaging neighboring tumor cells and enhancing the delivery of therapeutic siRNA. The results demonstrated effective decrease in tumor volume and prolonged animal survival when compared to liposomes lacking siRNA or absence of ultrasound treatment. 

### 4.2. Polymers

Cationic polymers are macromolecules that spontaneously bind DNA via electrostatic interactions. This unique property has been used commercially for cell transfection. They offer advantages such as small size and flexible chemistry that allows extensive modifications to improve biodistribution and tumor targeting.

A typical example of a linear polymer used to deliver plasmids or oligonucleotides is polyethylenimine (PEI). This polymer binds DNA strongly and has high transfection efficiency, forming small particles that enter the cells via endocytosis [185]. However, in absence of further modifications it has high cellular toxicity and cannot reach intracranial tumors when injected peripherally. Chemical engineering of PEI by addition of functional groups such as poly-ethileneglycol (PEG) or beta-cyclodextrin has proven sufficient to improve PEI permanence in circulation and in the tumor stroma [186]. PEI polymers modified by addition of myristic acid were able to cross the blood brain barrier, delivering a TRAIL-coding plasmid to intracranially implanted gliomas and increasing survival in tumor-bearing mice [187]. Similarly, PEGylated PEI was re-targeted towards glioma cells by chemical addition of an RGD-containing peptide [188]. This polymer (RGD-PEG-PEI) was injected intravenously and able to deliver TRAIL cDNA *in situ* in an intracranial glioma model, increasing animal survival.

In addition to the chemically simpler linear polymers, novel efforts have focused on using repeatedly branched polymers, known as *dendrimers*, for gene delivery. These molecules offer several advantages (such as high surface/volume ratio for DNA binding and well-known chemical behavior) [189] that have made them attractive synthetic nanocarriers for gene therapy. A commonly used dendrimer is a hyperbranched polymer of poly-amidoamine (PAMAM) characterized by biocompatibility, controlled biodegradation, low toxicity, and good accumulation in tumors with leaky vasculature [190]. A modified version of PAMAM was conjugated with nanoparticle carriers (see next section) and a viral Tat-peptide to facilitate cell membrane crossing [191]. This complex polymer (np-PAMAM-Tat) was used to deliver anti-EGFR shRNA to subcutaneously-implanted gliomas, inhibiting EGFR/Akt signaling and slowing tumor growth. Another modified version of PAMAM (Arg-PAMAM) has recently been used to deliver IFN-beta cDNA in intracranial glioma xenografts, causing selective tumor cell apoptosis and overall tumor shrinkage [192].

### 4.3. Nanoparticles

As their name indicates, these are nanometer-sized particles that, depending on their size (usually ranging from 20 to 50 nm in diameter) may spontaneously cross capillary walls and be endocytosed by cells. They have a rigid polymer core and a multi-functionalized surface that has been engineered to enhance DNA binding, particle diffusion, and cell-membrane crossing [193]. The core of the nanoparticles can also be modified with fluorochromes or metallic iron to facilitate *in vivo* imaging or to make them responsive to magnetic stimuli [194,195]. 

Super-paramagnetic iron oxide nanoparticles (SPIONs) have been used for delivery of therapeutic agents to the CNS while being at the same time tracked via magnetic resonance imaging [196]. In a recent study, SPIONs were covalently bound to the capsid of an RGD-targeted adenovirus (replication-deficient Ad5/3-RGD) and used to track the delivery of the virus [197]. Results demonstrated that viral infectivity of glioma cells was not affected by the SPIONs and that the virus could be tracked in a large (porcine) brain, but no studies were performed with gliomas *in vivo*. 

More complex version of SPIONs have used magnetic nanoparticles simultaneously functionalized on their surface with PEG (to bypass the blood brain barrier), chitosan (to improve half-life in circulation), PEI (to adsorb the therapeutic DNA) and chlorotoxin, a re-targeting peptide against glioma cells [198,199]. These multifunctional SPIONs have been used to demonstrate proof-of-principle delivery of siRNA and marker cDNA (eGFP) to glioma cells and subcutaneously-implanted gliomas.

### 4.4. Advantages and Challenges of Nanocarrier-Based Gene Therapy

Being completely synthetic, nanocarriers offer strong advantages as vehicles for gene therapy of GBM: size, structure and chemical composition of the carrier can be accurately tailored to enhance biodistribution, low toxicity, and optimal cell penetrance. The size of the DNA that can be carried is not as limited as in biological carriers, does not require prior genetic engineering of the carrier, and allows in principle any combination of plasmids and oligonucleotides as desired. The surface of nanocarriers is based on well-studied polymers that are biocompatible, biodegradable, and do not induce immune responses against the vehicle, as it is common when using viral carriers. As an additional advantage, the core of the carrier can also be optimized to track it using fluorescence or magnetic resonance, a property that would require additional modifications in viral or cell-based carriers. 

On the negative side, the major disadvantage of nanocarriers is that they are completely passive vehicles for gene delivery and their efficacy depends on the physical and chemical properties of the materials used to build them. Nanocarriers do not migrate actively and do not have neurotropism or even cell-specific tropism, therefore the tumor specificity must be “built from scratch” and optimized more exhaustively than with SCs or viruses. Their passivity as delivery vehicle also means that their distribution in the tumor will be much more limited than that observed with SCs and even with viral carriers, which can disperse when they lyse infected tumor cells. Finally, biological effects of novel particles and polymers (and their fragments) have not yet been analyzed in long-term studies and the overall efficacy of nanocarriers has not been exhaustively compared against other modalities for gene delivery. The future of these experimental vehicles for gene therapy of GBM will depend on these efficacy studies, using nanocarriers with improved cell type-specificity delivered via convection-enhanced delivery and combined with conventional therapies. 

## 5. Conclusions

Gene therapy represents today one of the more flexible and robust strategies for adjuvant therapy of GBM. As detailed in this review, gene-delivery approaches can be manipulated at multiple levels including choice of delivery vehicle, chemical or genetic engineering of the carrier, and selection of molecular targets, among others. This wide range of manipulations can be extensively exploited to optimize biodistribution, persistence, specificity, and targeting effects of the therapeutic agents, arguably to a much further extension that what can be achieved with improvements in conventional chemoradiotherapy. Optimized gene therapy approaches have repeatedly reached the clinical stage: A brief query of clinical trials listed by the Journal of Gene Medicine Clinical Trial website [11] and trials registered with the US National Institutes of Health [10] reveals, at the time of this writing, over a dozen active studies to deliver genetic material in malignant gliomas (Table 1). This underscores the extent to which gene therapy has become one of the most important approaches in molecularly-targeted therapy for GBM, and indicates that future developments in this field will continue improving the therapeutic options for this devastating disease. 
